# Autophagy in Age-Associated Neurodegeneration

**DOI:** 10.3390/cells7050037

**Published:** 2018-05-05

**Authors:** Athanasios Metaxakis, Christina Ploumi, Nektarios Tavernarakis

**Affiliations:** 1 Institute of Molecular Biology and Biotechnology, Foundation for Research and Technology-Hellas, Heraklion 70013, Crete, Greece; thanos_metaxakis@imbb.forth.gr (A.M.); ploumi@imbb.forth.gr (C.P.); 2 Department of Basic Sciences, Faculty of Medicine, University of Crete, Heraklion 70013, Crete, Greece

**Keywords:** ageing, Alzheimer’s disease, amyotrophic lateral sclerosis, autophagy, Huntington’s disease, mitophagy, neurodegeneration, Parkinson’s sisease, protein aggregation, treatment

## Abstract

The elimination of abnormal and dysfunctional cellular constituents is an essential prerequisite for nerve cells to maintain their homeostasis and proper function. This is mainly achieved through autophagy, a process that eliminates abnormal and dysfunctional cellular components, including misfolded proteins and damaged organelles. Several studies suggest that age-related decline of autophagy impedes neuronal homeostasis and, subsequently, leads to the progression of neurodegenerative disorders due to the accumulation of toxic protein aggregates in neurons. Here, we discuss the involvement of autophagy perturbation in neurodegeneration and present evidence indicating that upregulation of autophagy holds potential for the development of therapeutic interventions towards confronting neurodegenerative diseases in humans.

## 1. Introduction

Autophagy is an evolutionarily conserved physiological process that facilitates decomposition of unnecessary or dysfunctional cytoplasmic components and organelles in the lysosome. Depending on the substrate subjected to selective degradation, autophagy can, among other types, be categorized in ER-phagy (endoplasmic reticulum), mitophagy (mitochondria), ribophagy (ribosomes), lysophagy (lysosomes) and pexophagy (peroxisomes). Three main types of autophagy have been identified: macroautophagy, microautophagy and chaperone-mediated autophagy. Macroautophagy, commonly referred to as autophagy, requires the formation of an autophagosome, a double-membraned vesicle, in which proteins mainly, but also other macromolecules and organelles, are trapped prior to their degradation in the lysosomes. Under normal conditions, autophagy is active at the basal level but under various stresses, such as starvation, accumulation of misfolded proteins or protein aggregates, organelles’ damage and infections, it is rapidly stimulated [1].

Autophagy is a major contributor to intracellular homeostasis, since it selectively degrades cellular components that are unnecessary or even deleterious for cell survival. In starved organisms, autophagy prolongs survival through providing them with necessary energy and dietary components via recycling intracellular proteins and organelles. Several genetic studies, from yeasts to mammals, have identified more than 30 evolutionarily conserved autophagy-related genes (ATG), necessary for autophagy [2,3]. These genes are sequentially activated and facilitate the initiation, formation and elongation of the autophagosome, as well as its fusion with lysosome, for the generation of the autolysosome. Eventually, its contents are degraded and recycled. In general, autophagy has an essential role in the elimination of unnecessary, defective or aggregated proteins and damaged organelles, as well as in differentiation, cognition and development [4,5,6,7]. Hence, autophagy is a well-orchestrated, evolutionarily conserved mechanism through which organisms maintain intracellular homeostasis and especially proteostasis, and survive under adverse conditions [8].

Supportive of its role in homeostasis maintenance, a growing body of evidence from both human pathology studies and experiments with animal model systems has revealed a beneficial role of autophagy in organismal health. Several pathologies, including neurodegeneration, cancer, stroke and metabolic syndromes are associated with defects in the autophagic machinery [9,10,11]. Importantly, autophagy has been recognized as a major mechanism affecting healthspan and lifespan in animal model systems [12,13]. Proteostasis impairment through ageing, together with a simultaneous age-related decline in activity of ATGs, implicate autophagy and proteostasis in age-associated deterioration and disease [14,15].

Several reports find a strong association between ageing and downregulation or deregulation of autophagic activity [16,17]. Interestingly, autophagy is shown to be mainly regulated through metabolic pathways and food consumption that are shown to affect ageing and age-related diseases. Insulin/insulin-like growth factor-1 (IGF-1) signaling (IIS), the mammalian target of the rapamycin pathway (mTOR pathway), dietary restriction (DR) and starvation modulate both autophagy and longevity [18,19]. Importantly, genetic or pharmacological interventions that extend lifespan through a reduction of IIS, inhibition of mTOR, or a deacetylation of histones and DR also increase autophagy, while several studies suggest that their anti-ageing effects require autophagy. Moreover, several transcription factors involved in lifespan extension also contribute to the upregulation of ATG genes [20,21]. The above findings implicate autophagic impairment in the progression of age-related pathologies.

Loss of proteostasis is suggested to underlie several age-related neurodegenerative diseases, such as Alzheimer’s disease (AD) and Parkinson’s disease (PD), in which the gradual accumulation of protein aggregates causes neuronal impairment and death. The ratio of autophagosome formation to autophagic degradation controls autophagic flux, the deterioration of which causes neuronal cell death [22,23]. Neurons are post-mitotic cells, more easily affected by proteostasis impairment, since damaged proteins and organelles are not diminished by mitosis. Autophagy is important for the elimination of aggregated proteins and dysfunctional organelles. Hence, autophagy is a major proteostatic mechanism that could protect nerve cells from proteotoxicity. Indeed, autophagy impairment leads to neurodegenerative disorders [24,25] and the expression of several essential genes for autophagy are shown to be reduced in the brain through ageing, such as the *Atg5*, *Atg7* and *Beclin 1* genes [26]. Several studies show that autophagy decline in aged organisms might lead to neurodegeneration through impaired proteostasis and suggest genetic or pharmacological stimulation of autophagic components as putative therapies. However, overactivated autophagy is associated with exaggerated degradation of cellular components and neuronal cell death [27,28,29].

Here, we discuss the role of macroautophagy (herein referred to as autophagy) on the development of age-related neurodegeneration. We will present findings from research in animal model systems and humans supporting the assertion that dysfunctional autophagy and impaired proteostasis underlie, at least in part, the progression of the major neurodegenerative disorders. We also show evidence supporting the anti-ageing role of functional autophagy and we discuss the possible use of drugs enhancing autophagy for the treatment of age-related neurodegenerative diseases in humans.

## 2. General Autophagic Mechanisms

**Figure 1:**
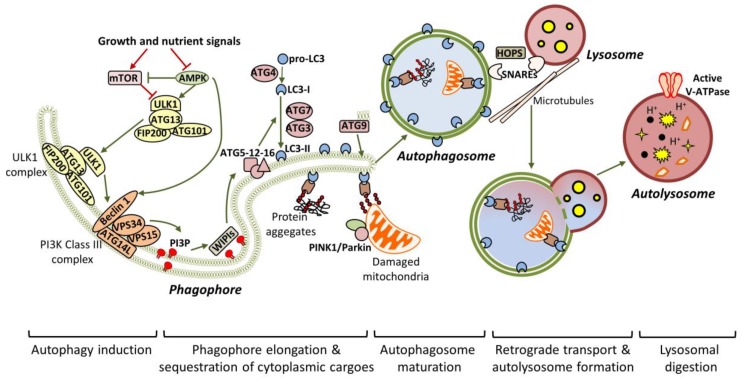
General autophagic mechanisms. Under nutrient-rich conditions, the autophagic pathway remains inactive due to mTOR-dependent phosphorylation of ULK1. Nutrient deprivation or stress, activate AMPK, which promotes autophagy initiation, through mTOR inhibition and concurrent ULK1 activation. Once active, the ULK1 complex is translocated to the pre-autophagosomal membrane structures (mainly derived by the endoplasmic reticulum (ER) or endosomal membranes), to initiate the formation of the phagophore. ULK1- and AMPK-dependent phosphorylation of Beclin 1 disrupt its interaction with anti-autophagic factors (like Bcl-2) and enhance the recruitment of the VPS34 complex. Subsequently, the active VPS34 complex acts as a PI3K to generate PI3P. The resulted PI3P interacts with WIPI proteins, which in turn promote the recruitment of autophagy-related gene (ATG) proteins, required for phagophore nucleation. The critical step for autophagosome formation is the conjugation of LC3/ATG8 to PE lipids, a reaction that requires the co-ordinated function of *ATG7*, *ATG3* and *ATG5-12-16* (acting as E1, E2 and E3 enzymes respectively). In parallel, ATG9 promotes autophagosome formation, as it provides lipids to the expanding phagophore. During expansion, specific adaptors sequester cytoplasmic material (like protein aggregates or damaged mitochondria) in the phagophore, by directly interacting with LC3-II. Scission events mediate the closure of the phagophore and the formation of the double-bilayered autophagosome. The newly-formed autophagosomes enter the microtubule-dependent retrograde transport to attach lysosomes. Autophagosome-lysosome attachment is aided by multiple proteins, including soluble NSF attachment protein receptors (SNAREs) and the scaffolding homotypic fusion and protein sorting (HOPS) complex. Following attachment, the outer autophagosomal membrane fuses with the single lysosomal membrane, resulting in the autolysosome. Efficient acidification (mediated by v-ATPase) activates the lysosomal hydrolases, which finally digest the sequestered cytoplasmic material.

Autophagy is a tightly-regulated process, which can lead either to the bulk degradation of intracellular material in response to increased energy demands, or to the selective elimination of specific cytoplasmic components in response to damage or stress. Nutrient deprivation and/or other stress stimuli, such as the accumulation of protein aggregates, commence a signaling cascade, which results in the formation of the isolation membrane, known as the phagophore, and its enclosure in the final autophagosome form [30] (Figure 1). Transcription factor EB (TFEB) is considered to be the master regulator of autophagy, since it regulates the expression of genes required for autophagosomal and lysosomal biogenesis [31]. Extensive studies in yeast have identified ATG proteins as the main factors that orchestrate the initiation and progression of autophagy. Most of these proteins are conserved in mammalian systems [32].

Mammalian ULK1/2 (homologous to yeast *Atg1* and *C. elegans* UNC-51) kinases pre-exist in the cytosol as a complex with ATG13 (homologous to yeast Atg13), focal adhesion kinase family interacting protein of 200kD (FIP200) and ATG101, and remain inactivated due to mTOR-dependent inhibition [33,34,35]. Upon starvation, the ULK1/2 complex is activated in an adenosine monophosphate-activated protein kinase (AMPK)-dependent manner and is specifically accumulated to endoplasmic reticulum (ER) membranes, possibly aiding in the formation of the pre-autophagosomal structure (PAS) [35]. In turn, the active ULK1/2 complex is crucial for the recruitment and activation of VPS34, the vacuolar protein sorting 34 homolog, which, in complex with VPS15, ATG14L and Beclin 1, acts as a class III phosphoinositide 3-kinase (PI3K) to generate phosphatidylinositol 3-phosphate (PI3P). Particularly, ULK1 phosphorylates Beclin 1 at *Ser14*, and this phosphorylation is enhanced by ATG14L and ultraviolet (UV) radiation resistance-associated gene protein (UVRAG), promoting either autophagy initiation or autophagy maturation respectively [36]. In addition to ULK1, AMPK mediates its pro-autophagic effects through Beclin 1 phosphorylation at T388 [37]. Both Beclin 1 and ATGL14L are required for the efficient recruitment of VPS34 to the PAS. WD-repeat protein interacting with phosphoinositides (WIPI) proteins detect the newly synthesized PI3P and function as a platform for the assembly of downstream ATG proteins, responsible for the elongation of the phagophore [38,39,40].

Two ubiquitin-like conjugation events are required for phagophore expansion. Firstly, the E1-like *ATG7* and the E2-like *ATG10* mediate the conjugation of *ATG12* to *ATG5*. The resulted complex is then associated with *ATG16L*, which is needed for the translocation of the entire complex to the phagophore. After *ATG4*-mediated cleavage, LC3/ATG8 is converted to LC3-I, which is finally conjugated to the lipid phosphatidylethanolamine (PE), giving LC3-II, in a process involving *ATG7*, *ATG3* and *ATG12-ATG5-ATG16L* (acting as E1, E2 and E3 respectively) [41,42]. ATG9 is an important regulator of autophagosome maturation, as it provides lipid components to the expanding phagophore [43,44]. During elongation, adaptor proteins such as P62/SQSTM1 (phosphotyrosine-independent ligand for the Lck SH2 domain of 62 KDa/sequestosome), OPTN1 (optineurin), NBR1 (neighbour of *BRCA1 Gene 1*) and NDP52 (nuclear dot protein 52 kDa) mediate the selective sequestration of ubiquitinated cytoplasmic contents (including organelles and aggregated proteins), by directly interacting with LC3-II [45,46]. After substrate sequestration, the phagophore finally closes in a process involving membrane scission [47]. Persistence of unclosed autophagic structures in *Atg2*-deficient cells supports the assertion that ATG2 is required for phagophore closure [48].

Once sealed, the autophagosomes are associated with microtubules and transported to the perinuclear region, where the lysosomes have already accumulated, in response to stress [49]. Subsequently, the autophagosomes locate in close proximity with lysosomes in a process involving the coordinated function of tethering factors, the most important of which are the SNAREs (soluble NSF attachment protein receptors) and the HOPS (homotypic fusion and protein sorting) complex [50,51]. Alternatively, the newly-generated autophagosomes can fuse with late endosomes to form amphisomes, before being engaged to lysosomes [52,53]. Notably, ATG4 seems to participate in the removal of LC3-II from the outer autophagosomal membrane, prior to lysosome fusion [54]. Following that, the outer autophagosomal membrane is fused with the lysosome’s single membrane to generate the autolysosome, in which the inner autophagosomal membrane and the engulfed material are broken down by lysosomal acidic hydrolases. Activation of the proton pump vacuolar-type H^+^ ATPase (vATPase) is a critical independent step for lysosomal digestion [55].

## 3. Autophagy and Age-Related Neurodegeneration

Ageing is the greatest risk factor for the most prevalent neurodegenerative diseases, such as AD and PD. With an ever-increasing human population and life-expectancy, modern societies are urgently seeking treatments for neurodegenerative diseases, while the number of people affected by neurodegeneration will increase in the near future. For example, the prevalence of AD is estimated to be duplicated within the next 20 years [56]. For this reason, understanding the mechanisms underlying age-related neurodegeneration is of great importance.

Impaired proteostasis is a common feature of most neurodegenerative diseases. Intriguingly, several studies show an association between neurodegeneration and alterations in the autophagic machinery. For instance, neurons of patients with AD [57,58] and PD [59] have a profound accumulation of autophagosomes. Interestingly, accumulated autophagosomes co-exist with aggregated proteins. This indicates that autophagosome formation and autophagic degradation are imbalanced, as well as the inability of autophagic machinery to eliminate aggregates in a coordinated way. Aggregated proteins can cause synaptic impairment, damage to organelles, and neuronal cell death. Other studies show that reduction of lysosomal acidification or decreased activity of lysosomal hydrolases is inhibitory for autophagic degradation and causes neurodegenerative diseases [6]. Also, inhibition of the microtubule-mediated autophagosomal and endosomal transport blocks the autophagosome–lysosome fusion that is associated with neurodegenerative diseases [60,61,62]. Indeed, recent studies directly link mutations in ATG genes with the development of neurodegenerative diseases such as AD, PD and amyotrophic lateral sclerosis (ALS) [16]. In addition, deletion of the essential autophagic genes *ATG7* [25] and *ATG5* [24] can sufficiently induce the formation of cytoplasmic inclusions and neurodegeneration in neurons.

In conclusion, experimental evidence indicates that autophagy impairment might underlie the progression of at least some neurodegenerative diseases. Here we will present findings from research in animal model systems supporting the association between autophagy function and progression of the most prevalent age-related neurodegenerative diseases affecting humans, PD, AD, Huntington’s disease (HD) and ALS.

### 3.1. Alzheimer’s Disease (AD)

AD is the most frequent type of progressive dementia diagnosed in elderly people worldwide. AD-affected brains are characterized by degeneration and eventual loss of neurons of the cerebral cortex and certain sub-cortical regions. The neuropathology of AD has been linked to the aberrant extracellular deposition of amyloid beta (Αβ) plaques and the intracellular formation of hyperphosphorylated tau-comprised neurofibrillary tangles (NFTs) [63].

Aβ peptide results from sequential proteolytic cleavages of the amyloid precursor protein (APP), an integral membrane protein mainly concentrated in synaptic regions. Human APP can be processed either by α-secretase, which releases the soluble APPα fragment, or by a β-site amyloid precursor protein-cleaving enzyme (BACE1), which releases the sAPPβ fragment. Cleavage by α-secretase is non-amyloidogenic, since it occurs inside the Αβ domain of APP. On the contrary, the β-secretase-processed membrane-associated APP fragment can subsequently be cleaved by γ-secretase, leading to the release of the pathogenic, aggregation-prone Αβ peptide [64,65,66]. Stress-induced expression of BACE1 has been suggested to be the initial step for AD pathogenesis [67,68]. Thus, age-related organismal changes could contribute to altered BACE1 expression and subsequent Αβ accumulation in the aged brain.

Tau is a microtubule-associated protein, ubiquitously expressed in the central nervous system. It has a crucial regulatory role in microtubule assembly and stabilization. In AD-affected brains, and other related tauopathies, like frontotemporal dementias (FTDs), tau is hyperphosphorylated and it self-aggregates, forming the intracellular NFTs [69,70]. Inability of the hyperphosphorylated tau to associate with tubulin results in the gradual disruption of microtubule network [71].

Electron microscopy studies have confirmed the presence of increased autophagosomes in post mortem human brain tissues of AD patients [57,58]. The increased number of autophagosomes is not correlated with induced autophagy, as previously thought, but rather reflects blockage in autophagic flux [72]. Aβ peptide can be generated intracellularly inside autophagosomes, in which APP, BACE1 and γ-secretase reside. The Αβ-loading of autophagosomes could lead either to the extracellular secretion of Αβ or to its lysosomal degradation. Studies have shown that autophagy is required for efficient Αβ secretion, since loss of the *Atg7* gene in APP transgenic mice results in reduced extracellular Αβ plaques and increased Αβ accumulation [73,74]. In healthy neurons, the newly formed autophagosomes follow the retrograde transport system along the axon to finally reach the soma, where the lysosomes are primarily located. Conversely, in AD pathology, the axonal vesicle transport is blocked, as shown in AD transgenic mice. In particular, Αβ oligomers interfere directly with amphisome transport by disrupting the dynein-Snapin complex [75]. Hyperphophorylated tau seems to be a contributing factor for defective retrograde transport [76].

In addition to the impairment in autophagosome transport, defects in lysosomal function have also been implicated in AD pathogenesis. Interestingly, presenilin 1 (PS1), one of the four factors that constitute the γ-secretase complex, is required for efficient lysosomal acidification. In particular, PS1 regulates the maturation and transport of v-ATPase, which is essential for the acidification of the newly synthesized autolysosomes. Deficits in lysosomal acidification lead to the accumulation of Aβ-loaded autophagosomes [77]. The fact that mutations in PS1 have been associated with the onset of familial AD has raised the intriguing question as to how loss of-function mutations in a protein required for Aβ generation can cause AD. Several studies have tried to give an explanation, providing contradictory results [78,79,80,81].

Overexpression of distinct Aβ peptides (Aβ42 and Αβ40) in *Drosophila* neurons uncovered an age-dependent neurodegeneration mechanism which likely involves disintegration of autolysosomal membranes, rather than defects in autophagosome maturation (transport and fusion). The authors suggested that the particular peptides can induce neurodegeneration due to leakage of lysosomal proteins from post-lysosomal vesicles [82].

Several studies have also implicated Beclin 1 deficiency in the pathophysiology of AD [83,84,85]. Specifically, neuronal expression levels of Beclin 1 drop physiologically during ageing but also in AD-affected brains [26,84,85]. Induced Caspase-3 activity in AD brain tissue is suggested to mediate the cleavage and the subsequent depletion of Beclin 1 [86]. A recent study showed that transgenic mice with a specific mutation in Beclin 1 (F121A) display constitutively induced autophagy in the brain (among other tissues) and significantly reduced Aβ accumulation. This mutation disrupts the interaction of Beclin 1 with its inhibitory partner, B-cell lymphoma 2 (Bcl-2) [87]. In addition to Beclin 1, several core autophagic genes in *Drosophila* (*Atg1*, *Atg8a* and *Atg18*), have been found to be downregulated in an age-dependent manner, leading to induced Αβ accumulation [88]. Nuclear receptor binding factor 2 (NRBF2), an important member of the PI3K complex, has been also shown to be significantly downregulated in the hippocampus of AD mouse models. In the particular study, they showed that NRBF2 positively regulates autophagy and is specifically involved in the autophagy-mediated degradation of APP C-terminal fragments, thus eliminating the generation of Aβ oligomers [89].

mTOR signaling is an additional autophagy-related pathway involved in AD pathology. Rat ovary cells, stably expressing a familial AD mutation, display increased mTOR signaling, as reflected by increased phosphorylation of its downstream targets, p70S6 kinase (p70S6K) and eukaryotic initiation factor 4E-binding protein 1 (4E-BP1) [90]. Subsequent studies have provided evidence that mTOR is also involved in tau-mediated pathogenesis in AD and related tauopathies. Increased mTOR activity induces tau expression and promotes its glycogen synthase kinase-3 (GSK3β)-dependent phosphorylation [91]. Additionally, mTOR promotes transportation of tau to exocytotic vesicles, facilitating its secretion in the extracellular space, as shown in AD-affected brains and in human neuroblastoma cells [92].

### 3.2. Parkinson’s Disease (PD)

PD is the second-most prevalent late-onset neurodegenerative disorder after AD [93]. Symptomatology includes tremors, muscular rigidity, akinesia, bradykinesia and postural instability [94]. These symptoms are caused by insufficient dopamine production in substantia nigra, due to the selective death of dopaminergic neurons [94,95,96]. Another characteristic of PD is the accumulation of ubiquitinated α-synuclein-containing inclusions within dopaminergic neurons. These inclusions are called Lewy bodies [96]. Accumulation of α-synuclein in the Lewy bodies is caused by impaired axonal transport [97].

There are two types of PD, the sporadic and the familial types, accounting for up to 90% and 10% of incidents, respectively [98]. In both cases, specific genetic mutations are involved in the disease progression. These genes are largely associated with the functionality of intracellular transport pathways that end up in the lysosome, hence implicating a putative involvement of autophagy in the aetiology of the disease [99]. Indeed, several findings suggest a blockade of autophagy in PD. Post mortem examination in brain samples from patients with sporadic PD show accumulation of autophagosomes, accompanied with loss of lysosomal markers in dopaminergic neurons [100,101]. Also, TFEB, an essential inducer of autophagy and lysosomal biogenesis [102], is absent from nuclei of dopaminergic neurons of PD patients, where it exerts its action [63]. These findings strongly implicate reduced autophagy in the aetiology of PD.

Dysfunctional mitophagy has been suggested to be a major cause of PD. Mitophagy is an essential mitochondrial quality-control mechanism and selectively eliminates damaged mitochondria. As such, loss of function mutations in two genes that are essential for mitophagy, the phosphatase and tensin homolog (PTEN)-induced putative kinase 1 (*PINK1*) and *Parkin*, cause sporadic juvenile-onset and autosomal recessive PD, respectively [103,104,105]. Mutations in these genes are the leading cause of parkinsonism [106]. Specifically, PINK1 is a kinase with a mitochondrial targeting sequence and a serine/threonine kinase domain and can be processed by presenilin-associated rhomboid-like (PARL), a mitochondrial protease, under normal conditions [107,108]. PARL-mediated procession of PINK1 leads to its degradation by the ubiquitin-proteasome system [109]. Under adverse conditions, such as mitochondrial depolarization, the processing of PINK1 by PARL is inhibited, thus causing its accumulation on the outer mitochondrial membrane. Subsequently, PINK1 autophosphorylates and recruits Parkin to damaged mitochondria [110]. Parkin is an E3 ubiquitin ligase which becomes phosphorylated and activated, together with ubiquitin [111,112,113], by PINK1. Although Parkin is not essential for mitophagy initiation, it enhances mitophagy. PINK1 phosphorylates ubiquitin which ubiquitinates mitochondrial surface proteins, thus leading to the formation of the phagophore around mitochondria and their targeting for lysosomal degradation. Mutations in the PINK1- and Parkin-expressing genes abrogate the above procedure and lead to mitophagy impairment that characterizes patients with PD. In support of this, the activity of essential autophagy genes are associated with the development of PD-like phenotypes in both flies and mice [114].

### 3.3. Huntington’s Disease (HD)

Another age-related neurodegenerative disease is HD. It is an autosomal-dominant neurodegenerative disease, characterized by the improper folding and neuronal aggregation of huntingtin (HTT) protein. Mutant HTT is generated by the cytosine-adenine-guanine (CAG) expansion, that encodes a polyglutamine (polyQ) at the N-terminus of HTT. Aggregation of HTT leads to neuronal death, causing movement disabilities, and mental and psychiatric pathologies [115,116,117]. Several mechanisms are implicated in HD-related neurodegeneration [118,119], due to the multifunctional role of HTT and its involvement in multiple cellular pathways [120]. Its structure is similar to three different autophagic proteins in yeast: Atg23, Vac8, and Atg11 [121,122] and it is essential for neuronal survival [123,124]. HTT has a regulatory role in autophagy through its interaction with ULK1 and p62 [125]. It induces autophagy by releasing ULK1 from mTOR, which inhibits the activity of the ULK1/2 complex. It also binds p62 and inhibits it from linking ubiquitinated substrates and LC3, thus leading to autophagy blockage.

In support of a role of HTT on autophagic mechanism, loss of polyQ in HTT stimulates neuronal autophagy [126], while recent data indicate that HTT enhances autophagy through acting as a scaffolding protein [121], thus facilitating substrate recognition through physical interaction with autophagic components [125]. Such a role of HTT is supported by experiments with cell lines, mouse models and cells derived from patients with HD, showing that expression of mutant HTT causes defects in cargo loading into autophagosomes [127]. Interestingly, the clearance of mutant HTT through pharmacological [128,129,130] and genetic [131] interventions attenuates neurodegeneration [128,130,131] and improves performance [128,129,130] in mouse models of HD. In conclusion, mutant HTT impairs autophagic function, while its autophagy-mediated clearance relieves neurons from toxicity.

### 3.4. Amyotrophic Lateral Sclerosis (ALS)

ALS is a fatal neurodegenerative disorder, characterized by the progressive loss of brain and spinal-cord motor neurons, and the subsequent atrophy of the voluntary muscles controlled by them. Although it was initially regarded as a juvenile disease, ALS seems to be age-related, since the incidence of disease is exaggerated with age [132,133]. The majority of ALS incidents are sporadic (approximately 90%), while only 10% are linked to genetic inheritance [134]. The exact molecular mechanisms leading to ALS pathology remain largely enigmatic. Despite the common neuromuscular defects of ALS patients, multiple factors that may contribute to ALS progression have been identified. The persistence of insoluble protein aggregates in the degenerating motor neurons reflects probable deficiency in the protein quality-control mechanisms, as evident in common neurodegenerative diseases [135,136].

Numerous studies have suggested the potential role of autophagy in ALS development [137,138,139,140]. Neuron-specific loss of Atg5 and Atg7 causes motor defects in mice, in the absence of any disease-related background, suggesting that impaired autophagosome formation may be the main contributing factor for the generation of toxic protein aggregates in ALS, among other neurodegenerative diseases [24,25]. Interestingly, mRNA and protein levels of both TFEB and Beclin 1 were found significantly reduced in transgenic mice expressing a mutant form of human superoxide dismutase 1, commonly aggregated in familial ALS (fALS) cases [141]. Mutated forms of superoxide dismutase 1 (SOD1) interact with Beclin 1 and inhibit its pro-autophagic activity in ALS mouse models. However, the same study provided contradictory evidence for a negative role of Beclin 1 in ALS, since Beclin 1 haploinsufficiency protects against the neurotoxic effects in SOD1-mutated ALS mice [142].

Another ALS-related factor implicated in autophagy regulation is the transactive DNA-binding protein 43 (TDP-43), the aggregated form of which has been observed in inclusions of almost all familial and sporadic ALS (sALS) cases. Sequestration of TDP-43 in cytoplasmic inclusions has been suggested to inhibit its main role as a regulator of mRNA stability. Atg7 mRNA is one of the TDP-43 targets found to be destabilized in mouse neuroblastoma cells subjected to TDP-43 knockdown [143]. Additionally, a recent study showed that loss of TDP-43 induces TFEB nuclear translocation and at the same time disrupts the autolysosome formation in human neuroblastoma cells [144]. Although contradictory, these observations strongly suggest that TDP-43 has a physiological role in autophagy regulation, and that its aberrant function may contribute to the autophagy-dependent degeneration of motor neurons.

An hexanucleotide expansion in the intronic region of the chromosome 9 open reading frame 72 (C9orf72) is the most common cause of ALS. The particular repeat-expansion has been suggested to perturb C9orf72 function [145]. C9orf72 seems to be an important autophagy regulator, as it has been reported that it directly interacts with the small GTPases Rab7 and Rab11, which assist in endo-lysosomal trafficking [146]. Importantly, recent studies indicated that C9orf72 acts as a guanine nucleotide exchange factor (GEF) for the activation of its interacting GTPases [147]. In addition to endolysosomal trafficking, C9orf72 has also been involved in the regulation of autophagy initiation, by facilitating the interaction between the ULK1 complex and Rab1a, an event required for the translocation of the ULK1 complex to the phagophore [148]. The particular interaction with Rab1a is independent of C9orf72’s GEF activity [148,149] .

Mutations in the selective autophagy receptors p62 and optineurin (OPTN) have been also linked with rare ALS cases [150,151]. Studies in motor neurons of ALS mouse models have shown that p62 can bind the mutant form of SOD1 in an ubiquitin-independent manner, leading to the sequestration of SOD1 aggregates in autophagosomes and their subsequent lysosomal degradation [152]. In addition to SOD1, p62 seems to be also involved in the autophagic elimination of TDP-43 aggregates [153]. Recent studies suggested that the L341V p62 missense mutation, identified in late-onset sALS patients, renders p62 unable to recognize and interact with LC3-II [154]. Interestingly loss of p62 in zebrafish led to motor neuron defects, similar to ALS symptoms [155]. Apart from p62, mutations in the ubiquitin-binding domain of OPTN were shown to inhibit autophagosome maturation, thus decreasing the elimination of HTT-positive inclusion bodies, as shown in transfected cell lines [156]. There have been characterized OPTN mutations which impair either its ubiquitin-binding activity (E478G) or its interaction with LC3-II (F178A) [157]. ALS-associated mutations in OPTN and TBK1 (TANK-binding kinase), its activating kinase, are suggested to cause mitochondrial dysfunction, by inhibiting the efficient sequestration of damaged mitochondria to autophagosomes [158,159]. Mutations in Valosin-containing protein (VCP) have also been identified in fALS cases [160]. VCP, alternatively known as p97, belongs to the AAA ATPase family and has been suggested to have a role in the selective degradation of ubiquitinated proteins through autophagy [161].

There have also been reported some ALS cases in which autophagosome trafficking is impaired. Specifically, mutations in dynein and dynactin strongly inhibit the transport of the autophagic vesicles in neuronal bodies of motor neurons, as shown in mice [162,163,164,165]. However, the ALS-related phenotypes observed in these cases could be a side effect, since dynein and dynactin regulate the transport of many different neuronal cargoes (including synaptic vesicles). In this regard, a recent study, indicated that dynein is required for the formation and maintenance of neuromuscular junctions, since it mediates the transport of acetylcholine receptors and muscle-specific tyrosine kinase (MusK) [166]. Dynein has also been found to be sequestered in SOD1-positive aggregates formed in spinal cord and sciatic nerve motor neurons of ALS mouse models. Interestingly, mutant SOD1 aggregates hijack the axonal transport machinery to be transported to the soma and mediate their neurotoxic effects [167].

The involvement of the chaperone system in macroautophagy also seems to be important for the clearance of pathogenic aggregates in ALS. Increasing evidence supports the assertion that the co-chaperone Bcl-2-associated athanogene 3 (BAG3) in a complex with heatshock protein 70 (HSP70) and Heat Shock Protein Family B (Small) Member 8 (HSPB8) participates in the transfer of mutant SOD1 peptides to aggresomes, thus enhancing their autophagic removal [168,169,170]. Interestingly, BAG3 is associated with p62/SQSTM1, which mediates the interaction with the LC3-II-marked phagophores [168,171]. Given that p62 is mutated in several ALS cases, as previously mentioned, the BAG3-HSP70-HSPB8 chaperone complex may be unable to stimulate the autophagic sequestration of pathogenic aggregates like those of mutant SOD1. Remarkably, the same chaperone complex has been also involved in the selective autophagic removal of mutant HTT, but as shown, its contribution is not sufficient [172,173].

## 4. Autophagy and Ageing

Although there is no clear evidence regarding the mechanistic link between ageing and neurodegenerative diseases, studies suggest that mitochondrial DNA mutations and oxidative stress are both causative agents for ageing and neurodegeneration [174,175]. Ageing is associated with proteostasis decline, reduced nutrient sensing, organelle and mitochondrial dysfunction, cellular senescence, and stem-cell exhaustion [176]. The main cellular mechanism maintaining proteostasis and mitochondrial dysfunction is autophagy. For this, it has been suggested that a decline in basal autophagy might underlie, at least in part, ageing [8]. Supportive evidence includes the downregulation of *Atg5*, *Atg7*, and *Beclin 1* autophagy genes that has been observed in aged human brains [26], as also the increased mTOR activity, accompanied by a decline in ATG protein levels in brains of aged rodents [177,178]. Such findings suggest decreased autophagic function in aged brains. In support, human skin fibroblasts of healthy individuals show age-related mitophagy decline [179,180], as also mitochondrial biogenesis reduction [181,182,183]. Mitochondrial DNA (mtDNA) content is reduced in the frontal cortex in aged rats, while mtDNA deletion content is increased [184]. The above, together with other findings [185], suggest that mitochondrial biogenesis and mitophagy during ageing are impaired.

Several findings indicate that functional autophagy is necessary for healthspan and enhanced longevity. Combined genetic, pharmacological and longevity studies in animal models associate impaired autophagy with reduced healthspan and lifespan [186]. In yeast, a short lifespan is associated with autophagy defects and autophagy mutants do not respond to lifespan extension under dietary restriction [94,187]. In *Caenorhabditis elegans*, mutations in essential ATGs cause lifespan shortening [95], while DAF-16/FOXO Controlled, germline Tumour affecting 1 (DCT-1) is important for mitophagy and longevity maintenance under stress [188]. In *Drosophila melanogaster*, lowered expression of ATG1 protein kinase and sestrin1, a negative feedback regulator of mTOR, shortens lifespan and causes mitochondrial dysfunction [189]. Also, rapamycin (mTOR pathway inhibitor) extends lifespan through autophagy in flies [190]. In mice, loss of essential ATGs lead to the accretion of inclusion bodies containing ubiquitinated proteins, lipofuscin-containing lysosomes and disorganized mitochondria [191].

Autophagy is not only a prerequisite for healthspan and enhanced longevity, but its enhancement can sufficiently extend lifespan. In flies, neuronal upregulation of Parkin increases lifespan, reduces levels of protein aggregation during ageing and modulates mitochondrial activity [114,192]. Several more examples show that autophagy induction through pharmacological treatment can promote longevity. Pharmacological perturbation of mTOR (autophagy inhibitor) through rapamycin treatment increases lifespan in all organisms tested [13]. Caloric restriction (CR), another intervention that affects metabolism and extends lifespan, prevents the downregulation of several autophagy effectors in aged rodents [178]. Also, drugs that alter the acetylation state of proteins are shown to affect autophagy and longevity. As such, both resveratrol and spermidine enhance autophagy by inhibiting proteins’ acetylation. The natural polyphenol resveratrol is an autophagy inducer that promotes lifespan. It is a deacetylase activator with its main target being *Sirtuin 1* (SIRT1), the mammalian ortholog of yeast *Sir2*. SIRT1 is a lifespan-extending NAD(+)-dependent protein deacetylase [193]. Regarding the mechanism of autophagy activation, resveratrol has been suggested to induce autophagy by decreasing protein acetylation [194], but evidence suggests that its action might be mediated through Death-associated protein kinase 1 (DAPK1) [195] and through mTOR inhibition [196]. Another drug that inhibits protein acetylation, the polyamine spermidine, extends the lifespan of yeast, worms and flies through increased autophagy [197]. Interestingly, spermidine enhances healthspan and lifespan by inhibiting EP300 (E1A-binding protein p300) acetyltransferase [198].

In conclusion, functional autophagy is a prerequisite for healthspan and enhanced lifespan in experimental animals, with existing evidence suggesting that functional autophagy has anti-ageing effects on health for its role on proteostasis maintenance. Given that the major risk for the development of age-related neurodegeneration in humans is ageing, but also that aggregate-prone proteins are used as autophagy substrates, such as tau [199], α-synuclein [200], mutant HTT [128], and mutant ataxin 3 [199], the usage of autophagy-enhancing drugs could be effective for treatment of neurodegenerative disorders in aged individuals.

## 5. Autophagy as a Therapeutic Target for Neurodegenerative Disorders

Autophagy upregulation is shown to have a neuroprotective and anti-ageing role in animal models. Its usage as a putative treatment of neurodegeneration, even if it cannot counteract the aetiology of each disease, could potentially improve the symptomatology of neurodegeneration, since it is known to promote cell survival, improve mitochondrial function and oxidative stress resistance. Interestingly, it has been reported for certain neurodegenerative disorders that abnormal proteins impede autophagy. Hence, general autophagy upregulation might improve pathophysiology [201,202,203].

Several drugs known to enhance autophagy are shown to decrease the accumulation of protein aggregates and improve neurodegenerative pathologies. In AD mouse models, long-term rapamycin treatment ameliorates symptoms, reduces Aβ42 (a main component of the amyloid plaques found in AD-affected brains), and rescues cognitive deficits [60,90]. On the other hand, autophagy induction after the formation of mature plaques and tangles was not effective on AD-like pathology or cognitive deficits [204] and rapamycin treatment of flies expressing Aβ1-42 was found to shorten lifespan [82]. Hence, it appears that the benefit of autophagy induction is context-dependent [205].

Nevertheless, rapamycin reduces huntingtin accumulation and cell death in HD cell models [128,206]. In addition, it has been shown to reduce aberrant protein aggregation in two models of ALS [207]. Despite the beneficial role of rapamycin on autophagy induction, it is also an immune suppressor and its chronic administration may cause unwanted side effects. For this, rapamycin analogues have been tested as alternatives to rapamycin. Carbamazepine (CBZ), an anti-epileptic drug that enhances autophagy by decreasing mTOR activity, has a similar role to rapamycin in transgenic mice models of AD. After three months of treatment with CBZ, cerebral amyloid plaque burden and Aβ42 levels were reduced, autophagy was enhanced, and AD pathologies were alleviated [208]. Another rapamycin analogue, temsirolimus, shows effects similar to rapamycin on AD models [209]. Finally, GTM-1, a novel mTOR-independent autophagy inducer, has been shown to ameliorate Aβ pathology in mice [210]. The small-molecule enhancer of rapamycin 28 (Smer28) has been also found to enhance the clearance of APP C-terminal fragments through stimulation of the Atg5-dependent autophagic degradation in cell lines and in rat primary neuronal cultures [211].

A promising drug for PD treatment is resveratrol, which induces autophagy through the AMPK/SIRT1 pathway, and has neuroprotective effects on a rodent model of PD [212]. A similar role has been observed for spermidine, another autophagy inducer [198]. Resveratrol is protective against dopamine toxicity for cells expressing mutant HTT. The relative mechanism is suggested to involve the rescue of ATG4-mediated autophagosome formation [213]. Another drug that is shown to affect autophagy through AMPK induction, Nilotinib, is shown to reverse loss of dopamine neurons and improve motor behaviour via autophagic degradation of α-synuclein in PD models [214,215]. Notably, it is also implicated in Aβ clearance [216]. Metformin is an additional AMPK-dependent agent that improves neurodegeneration in AD and HD mouse models [217,218]. The use of Berberine seems to be promising, since studies have reported its beneficial effects on AD, PD and HD mouse models [219,220,221]. Although its mechanism of action is not fully understood, it has been suggested that Berberine may promote autophagy through AMPK activation [222].

Trehalose, a natural disaccharide, is shown to reduce both accumulation of Aβ [223] and α-synuclein in vitro [224]. Moreover, it rescues dopaminergic activity in PD mouse models and delays pathology in HD mouse models [130,223,224]. Additionally, it has been shown to prolong lifespan in ALS mouse models [225]. Lithium is another promising drug for treatment of neurodegeneration, since it has been shown to facilitate the clearance of mutant α-synuclein in vitro [226]. Lithium treatment also suppresses tau protein in mice via GSK3β inhibition and reduces mutant huntingtin protein aggregates and cell death [227]. Moreover, lithium treatment improved neuronal survival and enhanced lifespan in an ALS model [228], but the beneficial effects seem to be sex-specific [229]. Finally, lithium also decreases the levels of inositol 3-phosphates (IP3), which inhibits autophagy [230]. Another inositol-lowering agent, Rilmenidine, is also shown to remove mutant huntingtin in cells [129,231,232]. Furthermore, mTOR-independent autophagy activators, such as calpastatin (calpain inhibitor), improve the removal of huntingtin and lower its toxicity in cellular HD models [233]. In this pathway, cAMP regulates IP3 levels, which in turn inhibit autophagy.

**Figure 2:**
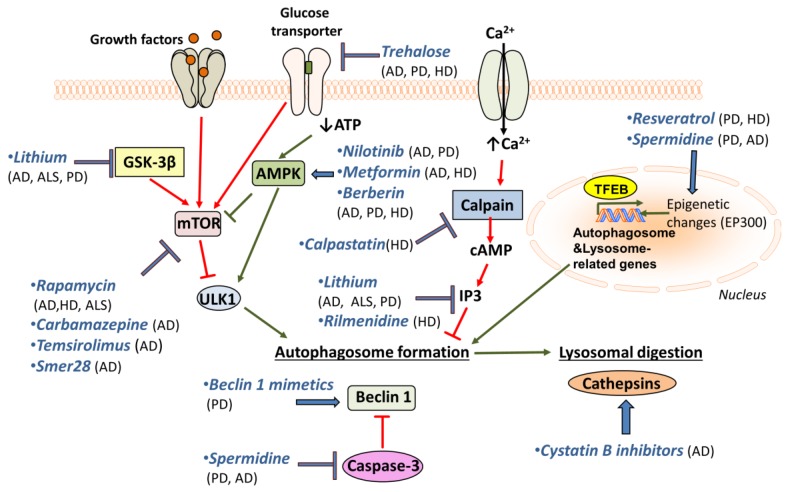
Putative pharmacological targets for autophagy enhancement. Selective pharmacological agents used to upregulate autophagy in model systems of neurodegenerative diseases.

In an attempt to increase autophagosome formation directly, intracerebral delivery of Beclin 1 in PD mouse models ameliorates the neurodegenerative effects [200]. This highlights the importance of using Beclin 1 mimetics or other autophagy inducers for the treatment of neurodegenerative diseases. Interestingly, the use of cystatin B inhibitors is promising, since it ameliorates cognitive decline in AD mouse models [234]. Cystatin B inhibits lysosomal activity by inhibiting cathepsins’ (lysosomal hydrolases) function. So, cystatin B inhibitors enhance lysosomal activity and thus promote the effective clearance of autophagosomes. The aforementioned drugs and their targets are shown in Figure 2.

## 6. Conclusions

Autophagy is an evolutionarily conserved mechanism that maintains intracellular homeostasis and promotes survival under adverse conditions. Impaired autophagy leads to the accumulation of intracellular toxic aggregates, harms healthspan, and decreases longevity. Recent evidence from research on humans but also on different animal model systems reveals a prominent anti-ageing role for autophagy. Specifically, autophagy is indispensable for the clearance of malfunctioning organelles, such as mitochondria, and aberrant protein aggregates that accumulate through ageing.

Several studies support the assertion that, at least in part, ageing is accelerated by the age-related autophagy decline that has been observed in several species, including humans. Also, functional autophagy is a prerequisite for lifespan extension achieved through different experimental interventions. Hence, autophagy decline might have a causal link to physiological deterioration observed in aged animals. Indeed, several studies suggest that functional autophagy delays ageing through maintenance of proteostasis.

Loss of proteostasis underlies the most prevalent neurodegenerative diseases in humans, such as AD and PD. Overall, increasing evidence indicates that deregulated autophagy plays a key role in the progression of neurodegeneration. Several studies on animal models of neurodegeneration reveal enhanced autophagy can decrease or even ameliorate intracellular accumulation of toxic aggregates and relative pathologies. These observations imply potential therapeutic strategies to prevent or treat neurodegenerative diseases in humans through pharmacological enhancement of autophagy. Regarding autophagy-enhancing agents, treatment dose and duration should be carefully chosen, as over-activation of autophagy could result in detrimental effects. Nevertheless, the existence of a large arsenal of autophagy-enhancing drugs is a valuable tool for the development of future treatments for neurodegenerative disorders.
